# Psychometric Properties and Development of a Scale Designed to Evaluate the Potential of Predatory Violent Behavior

**DOI:** 10.3389/fpsyg.2019.01648

**Published:** 2019-07-16

**Authors:** Julio C. Penagos-Corzo, Alejandra A. Antonio, Gabriel Dorantes-Argandar, Raúl J. Alcázar-Olán

**Affiliations:** ^1^Department of Psychology, Universidad de las Américas Puebla, Puebla, Mexico; ^2^Centro de Investigación Transdiciplinar en Psicología (CITPsi), Universidad Autónoma del Estado de Morelos, Cuernavaca, Mexico; ^3^Department of Psychology, Universidad Iberoamericana Puebla, Puebla, Mexico

**Keywords:** predatory violence, adolescents, suicidal tendencies, weapons, anger

## Abstract

The objective of this study was to develop and determine the psychometric properties of an instrument designed to detect traits and behavior that are associated with predatory violent behavior, which is defined as a determined, planned, controlled, and proactive aggression. The sample was comprised of 564 students, mostly in their last year of high school, or in their first year of college. The initial instrument had 78 items, ultimately resulting in 13 with good internal consistency (α = 0.825). Factor analysis showed four factors: anger-in, appeal for weapons, suicidal ideation, and the tendency to take justice into one’s own hands. Said factors showed significant correlations of convergent validity. Data shown here allows inferring that the instrument is a novel and concise tool that evaluates and detects the potential of predatory violent behavior.

## Introduction

According to the World Health Organization [WHO] (2002) violence is “the intentional use of physical force or power, threatened or actual, against oneself, another person, or against a group or community that either results in or has a high likelihood of injury, death, psychological harm, maldevelopment, or deprivation.” Thus, violence is characterized as (a) an intentional act that (b) involves force or power with the aim of (c) causing harm (Goetz, 2010). Violence has been classified in different ways: proactive, predatory, instrumental, affective, reactive, impulsive, and among others (Hanlon et al., 2013). However, all these categories can be identified in two large groups: affective (impulsive) and predatory (premeditated) (Siever, 2008). These modes of violence have different characteristics (Meloy, 2006). Affective violence is preceded by high levels of autonomic (sympathetic) arousal (Kockler and Meloy, 2007): is characterized by the emotions of anger and/or fear, and is a response to a perceived imminent threat. Its evolutionary basis is self-protection. In contrast, predatory violence is not preceded by autonomic arousal, is characterized by the absence of emotion and threat, and is cognitively planned. Its evolutionary basis is hunting for food (Meloy, 2006). In short, affective violence is more emotional and defensive, while predatory violence is planned, and it has the premeditated goal of attack (Raine et al., 1998; Ennis et al., 2017). It has been suggested that the differences between both types of violence may be greater if better methods of assessing them were available (Card and Little, 2006).

One of the violent juvenile behaviors that has been the focus of intervention programs is predatory violence (Elliot and Tolan, 1998). This violence is characterized by determined, planned, controlled, and pro-active aggression (Raine et al., 1998; Declercq et al., 2012), and it can include patterns of criminal behavior such as gang fighting (Ellickson et al., 1997) and even mass shootings (Declercq and Audenaert, 2011). Even though there appears to be no clear profile amongst those who perpetrate predatory violence, it has been observed that there are certain relevant elements that, while they do not clearly explain violent behavior separately, when found together may predict predisposition to such behaviors (Pollack et al., 2008).

Predatory violence has been related to several factors. These include biological (Ouellet-Morin et al., 2016; Klasen et al., 2018), psychological (Meloy, 2006, 2012; Declercq and Audenaert, 2011; Meloy et al., 2018; Capellan et al., 2019), and socio-structural explanations (Lee et al., 2014). Because psychological factors have consistently received empirical support, this study considered these factors, as they may represent solid constructs for assessing predatory violence.

The following variables explain and represent empirical evidence associated with this form of violence. In most cases of lethal violence in schools, perpetrators experienced acute social rejection beforehand (Leary et al., 2003). Such experiences involved mocking, intimidation, and even romantic rejection (Sommer et al., 2014). In this sense, there is experimental evidence indicating that people who feel socially rejected have a greater propensity to hurt others (DeWall et al., 2009). If high levels of anger emerge in the victim, there is risk of perpetrating extreme violence as a response. Actually, anger belongs to the stage of *gestation* (that is, the person experiments hate, resentment, hostility, humiliation, anger, and desires of revenge) of a violent act (Declercq and Audenaert, 2011). The next stage is consummation (Declercq and Audenaert, 2011), where the murders would then be the achievement of a desire of revenge for the injustice suffered by the perpetrator (Reuter-Rice, 2008; Pfeifer and Ganzevoort, 2017). Said consummation occurs when desires of revenge or taking justice by one’s hand are present (McCauley and Moskalenko, 2014), leading to violent justice. Moreover, the odds of violent acts increase with the availability and appeal for weapons or the proximity of firearms (Carlson et al., 1990; Killias and Haas, 2002; Newman et al., 2004; Newman and Fox, 2009; Monuteaux et al., 2015; Benjamin and Bushman, 2016; Benjamin et al., 2017; Emmert et al., 2018). In particular, additional evidence indicates a positive relationship between carrying weapons and school shootings (Dumitriu, 2013; Celis, 2015), homicides (Stroebe, 2013), and suicides (Burgess et al., 2006; Lankford, 2014, 2015).

Due to the diversity of factors present in violence, its prediction is a difficult challenge to address (National Collaborating Centre for Mental Health, 2015). However, assessing some of these factors may help to reduce the difficulty of their prediction (Andreu-Rodríguez et al., 2016). There are currently several instruments that evaluate aggressive behavior, such as the aggression questionnaire (AQ) by Buss and Perry (1992), the reactive/proactive aggression questionnaire for adolescents (RPQ) by Raine et al. (2006), the physical and verbal aggression questionnaire (AFV) by Caprara and Pastorelli (1993), the Latin-American multicultural inventory of the expression of anger and hostility (ML-STAXI) by Moscoso (2000), and among others. However, it appears that there is no instrument that measures the potential to execute an act of predatory violence. Evaluating this conduct is fundamental for its prevention and the protection of adolescents in general. Therefore, the objective of this study is to develop an instrument that evaluates traits and behavior associated with the risk of predatory violence in school environment. With regard to convergent validity, it is hypothesized that predatory violence will be moderately related to measures of aggression and antisocial behavior.

## Materials and Methods

### Participants

The initial sample was selected through availability and by invitation at selected schools. It was comprised of 598 students from educational institutions, both high school and college level, in the state of Puebla, in Mexico. However, some participants were removed from the sample due to the use of psychiatric substances (*N* = 12) that may be related to aggression: antidepressants (fluoxetine and lithium) (Cipriani et al., 2013; Molero et al., 2015) or anticonvulsant (carbamazepine) (Davico et al., 2018). Other participants were removed because their questionnaires were invalid (*N* = 22), according to a criteria based on MMPI2 items (Butcher et al., 2015), which will be explained later in the instruments. Therefore, the final sample was comprised of 564 students (152 high school students and 412 university students within the first four semesters of their studies). Of these, 60% were women and 40% men, with a mean age of 19 and a standard deviation of 1.76. From within this sample a sub-group of 269 participants was formed (62% women, 38% men) on which the instrument was tested and retested, within a time frame of 15–27 days. To guarantee the anonymity of the participants in the test-retest, when the pre-test was applied in a group, the participants were asked to write down the code assigned on the instrument in their notebooks. When the post-test was applied to the same group, they were asked to write the code in the questionnaire. Participant handling was carried out according to ethical standards established in the Declaration of Helsinki on research carried out on human beings (World Medical Association [WHA], 2013), guaranteeing anonymity, informed and voluntary consent, and absolute confidentiality. Each adult participant and legal tutor or parents for non-adult participant gave informed written consent prior to data collection. Informed consent from non-adult participants was given by parents or guardians at the educational institutions where the study was conducted. The study protocol received ethical approval from the Ethics Committee which is part of Universidad de Las Américas Puebla Research Committee (UDLAP Research Committee).

### Instruments

#### Potential Predatory Violence Inventory

This instrument (13 items) evaluated the presence of indicators that are associated with behaviors of predatory violence. Participants were required to choose to what extent each item described themselves on a 4-point Likert scale (1 = not at all, 4 = very much), where a higher score reflected (behaviors indicative of / associated with) predatory violence. The instrument was written and presented in Spanish as it was the native language of the participants.

#### Aggression Questionnaire (AQ)

This instrument (Buss and Perry, 1992) evaluated several aspects of aggressive behavior in the general population. It has 29 items (α = 0.88) distributed in four subscales: (1) physical aggression (α = 0.82), verbal aggression (α = 0.77), wrath (α = 0.67), and hostility (α = 0.75). The instrument evaluated aggression on a 5-point Likert scale (1 = extremely uncharacteristic of me, 5 = extremely characteristic of me), so that a higher value resulted in higher aggression. This instrument has been tested in reliability and validity on Mexican populations (Pérez et al., 2013).

#### Dissocial Behavior Scale (ECODI27)

This scale, with 27 items, evaluates dissocial behavior, which is defined as “behavior that precedes antisocial personality disorder and severe problems with the law” (Moral and Pacheco, 2011, p. 199). The scale has a high internal consistency (α = 0.91) and is structured around six factors: (1) theft-vandalism (α = 0.88), (2) mischief (α = 0.77), (3) school dropout (α = 0.83), (4) brawls and weapons (α = 0.78), (5) graffiti (α = 0.72), and (6) defying oppositionist behavior (α = 0.69). The instrument evaluated this variable on a 5-point Likert scale (1 = totally agree, 5 = totally disagree), where a higher score indicated a lower presence of dissocial behavior.

### Procedure

**FIGURE 1:**
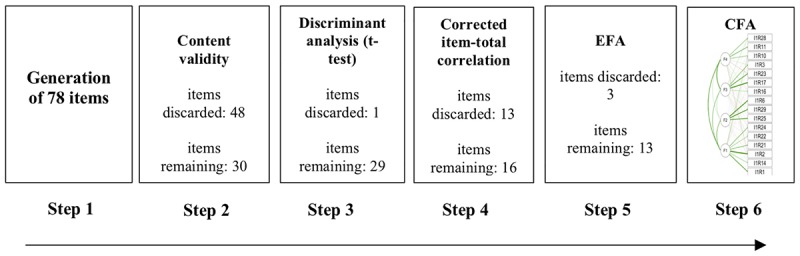
Steps in item selection process.

The initial number of items was 78 -new, elaborated and drew up for this study-, which followed standards established for the construction of instruments (Cohen and Swerdlik, 2001; Kaplan and Saccuzzo, 2012). Said items were developed by answering the question: “What are the indicators present in predatory violence?” Based on the literature (Anderson and Bushman, 2002; Leary et al., 2003; Pollack et al., 2008; Declercq and Audenaert, 2011; Monuteaux et al., 2015; Bushman et al., 2016; Gerard et al., 2016; Freedman et al., 2017; Paradice, 2017; White, 2017), items on anger, appeal for weapons, resentment, taking justice into one’s own hands, suicidal ideation, substance abuse, bullying, and a sense of belonging in school, were generated. Content validity was obtained through a panel of evaluators that determined if items (e.g., “Having a weapon makes me feel more secure,” or “I have had feelings of revenge”) were essential to the construct of predatory violence. Specifically, to test content validity, six judges were informed about predatory violence providing examples of aggression and violence (Anderson and Bushman, 2002), and they were provided with an abstract of about 50 words on evidence related to predatory violence. Judges were then required to evaluate the importance of said items using a 3-level criteria: (1 = the item is essential, 2 = the item is useful but not essential, and 3 = the item is not essential) (Lawshe, 1975; Tristán, 2008). Following Lawshe (1975) and Tristan’s algorithm (2008), items that had values below 0.75 were discarded. As a result, 26 items were maintained. Four items about interaction with peers and belonging to the educational institution were kept because of their relevance in the literature (Leary et al., 2006; Baird et al., 2017). The value with Tristan’s algorithm was above 0.66 in these items. The result was a 30-item scale. Figure 1 illustrates the procedure for selecting items to reach the final scale.

Due to the fact that the instrument handles a sensitive issue, the scale was headed “You, in your world,” intending to reduce social desirability or “normalize” the items. In addition, four items to detect social desirability and random filling of surveys were included. Three were selected from the MMPI (Butcher et al., 2015): (1) “Once in a while I think of things too bad to talk about,” (2) “I do not always tell the truth,” and (3) “I think nearly anyone would tell a lie to keep out of trouble”), and one was written based on the type of items observed on the *K* scale of correction: “I’ve forgotten how to write.” The first three items are considered critical to validity in the MMPI2 (Butcher et al., 2015) and the latest, which allowed detecting the random filling out of surveys. Contradictory responses to these items led to invalidating the potential predatory violence inventory (PPVI) for these participants.

A pilot study was administered on 100 participants. This led to grammatical and semantical corrections, and the final version included information relevant to participants, such as age, gender, educational level, and consumption of controlled substances. Finally, the PPVI was administered in combination with the ECODI27 and the AQ. Test-retest evaluation was carried out to determine temporal stability in a subsample (*n* = 269), in an interval of 15–27 days (Anastasi and Urbina, 1997). This study combined empirical-statistical and rational-theoretical criteria to select the items of the final scale based on this rationale. One of the underlying assumptions considered for the exploratory factor analysis (EFA) was to include all of the items simultaneously. Although statistical or theoretical criteria may lead to retain or reject items, it is possible to rely on one’s own decisions on reaching a parsimonious solution (Hair et al., 2014) that made logical and credible sense of the data. In this logic, “…choosing the number of factors is something like focusing a microscope. Too high or too low and adjustment will obscure a structure that is obvious when the adjustment is just right. Therefore, by examining a number of different factor structures derived from several trial solutions, the researcher can compare, and contrast to arrive at the best representation of the data” (Hair et al., 2014). This pointing guided the selection of items and the determination of factors.

### Statistical Analyses

Statistical analyses were carried out on the SPSS 23, except the confirmatory factor analysis (CFA) which was carried out on AMOS 18. A student’s *t* was used to evaluate if items discriminated correctly. The purpose of this analysis was to evaluate the discrimination capacity of each item, according to the levels of violence (high vs. low) reported by the instrument. Then, reliability was established through an internal consistency method and temporal stability, using Cronbach’s Alpha for the former and Pearson’s correlation for the latter. Correlations were interpreted with Cohen (1988), where values below 0.10 are considered trivial, between 0.10 and 0.30 are considered medium, and above 0.50 is considered large. As a general criterion for incorporating items into the EFA, it was considered that items in Cronbach’s alpha analysis yielded 0.30 or more in the corrected item-total correlation. An EFA was carried out on the scale, using principal axis factoring (PAF) and promax rotation of axes due to the conceptual consideration that the underlying factors may be correlated. For the factor extraction process, the main criteria were: eigenvalues higher than 1 and factor loadings higher than 0.40. The confirmatory factor analysis was assessed with the Comparative fit index (CFI) and the Tucker-Lewis index (TCI), where values in the range of 0.90–0.95 indicate acceptable model fit (Brown, 2006; Awang, 2012); and with the root mean squared error of approximation (RMSEA), where values below 0.05 indicate good fit (Brown, 2006; Awang, 2012). The sample size (*n* = 564) was randomly split in two groups, distributing approximately the same proportion of males and females to both samples. The first sample (Sample A) served to conduct the EFA, whereas the second one (Sample B), was used for the CFA. Sample A had 364 participants (40% males, 60% females) because 300 cases is good for factor analysis (Tabachnick and Fidel, 2007). Also, the size is adequate because it is within the standard of 10 individuals per item (Hair et al., 2014). Sample B had 200 participants (40% males, 60% females). This sample size is also adequate, based on the requirement of 10 participants per item (Bentler and Chou, 1987). Finally, correlations with the other instruments were established through Pearson’s correlations.

## Results

### Item Analysis

Discriminant analysis for items was carried out using two groups of participants comparing the highest scores against the lowest (i.e., the top and bottom 25%, respectively). A student’s *t* for independent samples was drawn, achieving a level of significance of <0.05. There was only one item that had to be removed: “weapons make me nervous,” because it did not show significant differences between groups.

### Reliability

#### Internal Consistency

An early internal consistency analysis consisting of a Cronbach’s Alpha showed an acceptable value (α = 0.772). However, several items yielded values less than 0.3 in item correlation (Table 1). These items (4, 5, 7, 9, 12, 15, 18, 19, 20, 26, 31, 32, and 34) were eliminated (see Figure 1). The resulting version had 16 items that yielded good internal consistency (α = 0.825). Final internal consistency, after eliminating three more items based on EFA yielded a 0.794 for all factors together, and 0.692, 0.695, 0.797, and 0.646 for, respectively, Factors 1, 2, 3, and 4.

**TABLE 1:** Correlation weights for final items.

**Items**	**Corrected item-total correlation**	**Alpha if item deleted**
1. I have felt the need for revenge/*He sentido deseos de venganza*	0.527	0.752
2. I keep resentments that I don’t share with anybody/*Guardo rencores que no comento a nadie*	0.451	0.757
3. I like using weapons that look very real/*Está padre utilizar armas que simulan ser de verdad*	0.317	0.764
4. I vent my anger on others*/Termino sacando mi enojo con los demás*	0.216	0.769
5. I think it’s bad that some of my classmates are bullied/*Creo que es malo que mis compañeros sufran acoso escolar/bullying*	−0.030	0.784
6. I have wished I didn’t exist*/He deseado dejar de existir*	0.385	0.760
7. I am good at adapting to others*/Soy bueno adaptándome a los demás.*	0.148	0.773
9. I have a hard time forgetting other people’s criticism/Me cuesta trabajo olvidar una crítica hacia mí	0.282	0.766
10. I have fantasized about taking justice into my own hands*/He fantaseado con la posibilidad de hacer justicia por mi propia mano*	0.618	0.745
11. I could sacrifice my life for an ideal/*Podría sacrificar mi vida por un ideal*	0.328	0.763
12. I have used real weapons*/He usado armas de verdad*	0.233	0.768
14. I have been treated unjustly*/Me han tratado injustamente*	0.397	0.759
15. I drink alcoholic beverages*/Consumo bebidas alcohólicas*	0.149	0.774
16. I have planned actions to take justice into my own hands with friends/*He planeado con amigos o conocidos acciones para hacer justicia por mi propia mano*	0.357	0.764
17. I would like to hold a real weapon*/Me gustaría tener contacto con armas de verdad*	0.416	0.758
18. I would sacrifice my life for a belief/*Sacrificaría mi vida por una creencia*	0.244	0.768
19. I have done things to animals that others see as torture*/He hecho cosas a animales que otros ven como tortura*	0.174	0.770
20. I have had fun watching my classmates bully another classmate*/Me he divertido viendo a mis compañeros hacerle bullying a otro*	−0.263	0.788
21. I have kept my anger bottled up inside*/Me he quedado con mi enojo*	0.333	0.763
22. I suspect that there are people who threaten my plans/*Sospecho que hay gente que amenaza mis planes*	0.466	0.757
23. Having a weapon makes me feel safe or confident/*Tener un arma me da seguridad o confianza*	0.400	0.760
24. I have lost control because of my anger*/He perdido el control por estar enojado*	0.504	0.752
25. I have found myself thinking about hurting myself*/Me he descubierto pensando en hacerme daño*	0.477	0.758
26. I have been glad to observe bullying*/Me he alegrado al presenciar acoso escolar/bullying*	−0.264	0.782
28. I enjoy movies and videogames where people take justice into their own hands/*Disfruto de películas y videojuegos donde se hace justicia por propia mano*	0.414	0.757
29. Sometimes, suicide is an option for me*/A veces, el suicidio para mí es una opción*	0.394	0.763
31. I am good at adapting to my environment/*Soy bueno adaptándome a mi entorno*	0.174	0.771
32. I use illegal substances*/Consumo drogas no legales*	0.231	0.768
34. I feel like a part of my educational institution/*Me siento parte de mi institución educativa*	0.112	0.775

#### Temporal Stability

To assess temporal stability of the instrument, a Pearson’s *r* was calculated for the test-retest (*r* = 0.693, *p* < 0.001). This was done with a sub-sample (*N* = 269) obtained by availability of participants, with an interval of 15–27 days. Statistical analysis was carried out with 13 items which was the final number of items.

### Construct Validity

#### Exploratory Factor Analysis

An exploratory factor analysis was carried out on Sample A using principal axis factoring and oblique rotation (Promax). Sample adequacy showed a KMO of 0.828, and Bartlett’s Sphericity Test resulted significant (*X*^2^_120_ = 1606.217, *p* < 0.001), which indicated that it was pertinent to carry out the factor analysis. The criteria extraction was eigenvalue >1.0 (Kaiser, 1960). In addition parallel analysis (Horn, 1965) was carried out [*X*^2^ (62) = 124.257, *p* < 0.001]. The EFA yielded a four-factor structure (Table 2), both with Kaiser’s criteria (eigenvalue >1.0), and with the parallel analysis. The first factor yielded an eigenvalue of 4.552 and explained 28.45% of total variance, the second factor had an eigenvalue of 1.962 and explained 12.27% of total variance, whilst the third had a value of 1.477 and explained 9.23% of total variance, and the fourth factor had an eigenvalue of 1.029 and explained 6.43% of total variance. All four factors explained 56.38% of total variance. Item 22, 14, and 16 had a factor loading of less than 0.4, and therefore it was eliminated, which resulted in a 13-item scale as a final version.

**TABLE 2:** Factor loadings with a four solution.

**Items**	**Factors**			
	**F1**	**F2**	**F3**	**F4**
2. I keep resentments that I don’t share with anybody	**0**.**863**	0.052	−0.123	−0.261
1. I have felt the need for revenge	**0**.**659**	0.032	−0.046	0.042
24. I have lost control because of my anger.	**0**.**514**	−0.160	0.095	0.183
21. I have kept my anger bottled up inside	**0**.**495**	−0.131	0.092	0.053
22. I suspect that there are people who threaten my plans	0.389	0.199	0.144	−0.030
14. I have been treated unjustly	0.378	−0.075	0.166	0.157
17. I would like to hold a real weapon	0.135	**0**.**803**	0.026	0.016
23. Having a weapon makes me feel safe or confident	0.123	**0**.**666**	0.076	−0.209
3. I like using weapons that look very real	−0.158	**0**.**512**	0.037	0.193
16. I have planned actions to take justice into my own hands with friends	0.112	0.295	−0.098	0.213
6. I have wished I didn’t exist	0.040	0.066	**0**.**807**	−0.032
29. Sometimes, suicide is an option for me	−0.024	0.155	**0**.**725**	−0.088
25. I have found myself thinking about hurting myself	0.044	−0.002	**0**.**673**	0.141
11. I could sacrifice my life for an ideal.	−0.119	−0.057	0.038	**0**.**562**
28. I enjoy movies and videogames where people take justice into their own hands	0.007	0.269	−0.077	**0**.**488**
10. I have fantasized about taking justice into my own hands	0.340	0.149	−0.052	**0**.**445**

*Salient and highest loadings per item in bold.*

#### Confirmatory Factor Analysis

The solution was submitted to a CFA on Sample B. The correlation’s matrix was studied through the maximum likelihood estimation method. The model fit indexes suggested an adequate fit for the 4-factor solution, χ^2^(57) = 1.413, *p* < 0.05, with optimal levels TLI = 0.953, CFI = 0.965, and RMSEA = 0.046. In addition, since men tend to have higher levels of violence than women, a CFA comparison was made using critical ratios for differences between parameters, in order to see if the scale worked similarly, for both genders, or if it had differences. The results of the analysis indicate significant differences only in item 3 of factor 2 (CR males = 0.984, CR females = 0.283; *z* = 2,766, *p* < 0.001).

#### Convergent Validity

The correlation with Buss and Perry’s AQ (1992) yielded a significant value (*r* = 0.609, *p* < 0.001), whilst the correlation with the ECODI27 (Moral and Pacheco, 2011) yielded a negative correlation (*r* = −0.519, *p* < 0.001). Table 3 illustrates the correlations between factors and the different scales that were used. The total scores of the three scales (PPVI total, AQ Buss-Perry total and Dissocial total) correlated above 0.50 (Table 3), which represents large correlations according to Cohen (1988). This result indicates convergent validity (Cohen and Swerdlik, 2001), particularly between the total score of the PPVI, and previous scale.

**TABLE 3:** Pearson′s correlations amongst variables and factors included in this study.

**Scales**	**Anger-in**	**Appeal for weapons**	**Suicidal ideation**	**Taking justice into one′s own hands**	**Total PPVI**
Appeal for weapons (PPVI)	0.249^∗∗∗^				
Suicidal ideation (PPVI)	0.350^∗∗∗^	0.195^∗∗∗^			
Taking justice into one’s own hands (PPVI)	0.425^∗∗∗^	0.496^∗∗∗^	0.193^∗∗∗^		
Total PPVI	0.765^∗∗∗^	0.673^∗∗∗^	0.566^∗∗∗^	0.781^∗∗∗^	
Physical aggression	0.500^∗∗∗^	0.379^∗∗∗^	0.254^∗∗∗^	0.446^∗∗∗^	0.577^∗∗∗^
Verbal aggression	0.398^∗∗∗^	0.169^∗∗∗^	0.099^*^	0.322^∗∗∗^	0.374^∗∗∗^
Wrath (Buss-Perry)	0.553^∗∗∗^	0.103^∗∗^	0.244^∗∗∗^	0.231^∗∗∗^	0.424^∗∗∗^
Hostility	0.498^∗∗∗^	0.120^∗∗^	0.375^∗∗∗^	0.222^∗∗∗^	0.440^∗∗∗^
Total (AQ, Buss-Perry)	0.645^∗∗∗^	0.267^∗∗∗^	0.329^∗∗∗^	0.409^∗∗∗^	0.609^∗∗∗^
Theft and vandalism	−0.234^∗∗∗^	−0.302^∗∗∗^	−0.103^*^	−0.225^∗∗∗^	−0.312^∗∗∗^
Mischief	−0.330^∗∗∗^	−0.378^∗∗∗^	−0.112^∗∗^	−0.396^∗∗∗^	−0.446^∗∗∗^
School dropout	−0.188^∗∗∗^	−0.205^∗∗∗^	−0.252^∗∗∗^	−0.131^∗∗∗^	−0.267^∗∗∗^
Brawling and weapons	−0.367^∗∗∗^	−0.404^∗∗∗^	−0.074	−0.416^∗∗∗^	−0.467^∗∗∗^
Graffiti	−0.132^∗∗^	−0.239^∗∗∗^	−0.094^*^	−0.143^∗∗∗^	−0.205^∗∗∗^
Defying behavior	−0.321^∗∗∗^	−0.191^∗∗∗^	−0.089^*^	−0.318^∗∗∗^	−0.344^∗∗∗^
Dissocial total	−0.400^∗∗∗^	−0.439^∗∗∗^	−0.158^∗∗∗^	−0.427^∗∗∗^	−0.519^∗∗∗^

^∗∗∗^Correlation is significant at level 0.001. ^∗∗^Correlation is significant at level 0.01. ^*^Correlation is significant at level 0.05.

#### Normative Data

Normative data of the sample (*N* = 564) indicated a mean of 8.28 and a standard deviation of 2.35 for F1; F2 yielded an *M* = 4.22, *SD* = 1.78; F3, *M* = 4.05, *SD* = 1.56; F4, *M* = 5.48, *SD* = 2.16, and Total PPVI *M* = 22.02, *SD* = 5.56. Derived from literature it is found that there are differences amongst gender groups for aggression and violence, so group differences were drawn. These can be found in Table 4.

**TABLE 4:** Gender comparisons of the PPVI values.

**Scales**	**Men**				**Women**				***t***
	***M***	***SD***	**Skewness**	***Kurtosis***	***M***	***SD***	**Skewness**	***Kurtosis***	
Anger-in (F1)	8.69	2.49	0.43	0.04	8.00	2.21	0.73	0.81	3.44^*^
Appeal for weapons (F2)	5.10	2.08	1.06	0.54	3.64	1.26	2.65	7.93	10.43^*^
Suicidal ideation (F3)	3.96	1.70	2.23	4.74	4.10	1.46	1.51	2.09	−0.98
Taking justice into one’s own hands (F4)	6.77	2.23	0.35	−0.44	4.62	1.62	1.21	1.31	13.2^*^
Instrument total	24.53	6.00	0.66	0.29	20.36	4.56	1.02	0.95	9.34^*^

^*^¡0.001.

## Discussion

Data here presented show the existence of four factors related to potential violent predatory behavior in a school environment. F1 can be identified as “anger-in,” F2 as “appeal for weapons,” F3 as “suicidal ideations,” and F4 as “taking justice into one’s own hands.” Furthermore, the instrument here provided appears to possess acceptable psychometric properties. The presence of anger, in combination with feelings of revenge, can be motivators for assailants that wish to take justice into their own hands (McCauley and Moskalenko, 2008, 2014, 2017), which is parallel to those reported in this study. Anger, as much as the desire for revenge, expressed as the desire to take justice into one’s own hands, shows a high correlation rate according to our data. This evidence is also found in Paradice (2017), who finds that the principal motivation for mass murders is anger. In this study, anger is the factor that most variance explains according to our scale, which makes sense given the correlation between suicidal ideation and contained anger, as factors in the inventory.

Our findings bind in a single scale some of the psychological variables that have been reported in literature as causal or related to predatory violent behavior. The instrument here developed reveals those behaviors or traits that could be connected, potentially, to the perpetration of violent acts. This may allow specialists to carry out early interventions that may avoid sinister acts of extreme magnitude that put the lives of young people in educational institutions at risk. Researchers in basic and applied psychology, as well as clinical and educational psychologists may be these specialists. Psychological research can provide useful information to prevent access to weapons by children and adolescents. For example, it has been reported that there is a strong association between gun ownership in the home and adolescent suicide (Knopov et al., 2019). It is therefore necessary to implement greater security measures if an assessed person has high values in liking weapons and suicidal ideation. In this regard, evidence indicates that laws punishing adults who leave weapons in unsafe places have contributed, albeit modestly, to the reduction of adolescent suicides and involuntary shootings by children (Webster et al., 2004). On the other hand, forms of psychological support should be sought by people who have high levels of anger or desire to take justice into one’s own hands. For example, a study of 156 adolescent offenders found that a violence prevention program significantly reduced anger and aggression and produced improvements in self-control (Zhou et al., 2018).

Data provided by the present study coincide with other findings on gender differences reported in the literature (Buss and Perry, 1992; Anderson and Bushman, 2002; Lankford, 2015), which is why it is recommendable to repeat this same study on a larger sample that includes as many men and women as possible in an effort to establish possible differences in the alignment of factors. Another suggestion is to carry out a test of validity on actual perpetrators of predatory violence.

### Limitations

The temporal stability of PPVI was evaluated at a fairly short interval. This represents a limitation. However, as a first approach to temporal stability, the test-retest correlation was relatively satisfactory (*r* = 0.693). Upcoming studies might evaluate the PPVI in a longer interval, but shorter than 6 months. Intervals of more than 6 months may include both a wider area of behaviors than the behaviors covered by the test, as well as random effects (Anastasi and Urbina, 1997). Another limitation of the work is the reliability coefficients achieved. These coefficients are satisfactory because in addition to showing a moderate level (Vera-Jiménez et al., 2014), the instrument has few elements (Jackson and Verberg, 2006), and is the first finished version of the test (Nunnally and Bernstein, 1994). However, it is necessary to find a way to increase reliability later, for example by adding more items.

School populations might not be the most adequate samples for the study of violent behavior. However, youngsters that belong to this population have been known to display violent behavior against their peers, and said behavior has been known to be lethal, both inside, and outside school contexts. Another limitation is that there was no evaluation of planned, controlled, and proactive aggression. The detection of these predatory violent behaviors would have strengthened the evaluation of the PPVI. However, said information was collected in an indirect manner, through other scales that measured the involvement in brawls and the use of weapons, vandalism, hostility, physical and verbal aggression, and wrath. In either case, the PPVI correlated with the other scales here compared in the direction expected, which allows assuming certain potential and usefulness of the presented instrument.

Another essential aspect that was not evaluated, and should have been, is predictive validity. Future research should strive to identify income of university level students, especially in people that score high on the PPVI. Said individuals should be followed up on in an effort to identify the presence of violent predatory acts, or those that are of an aggressive nature.

Finally, since violence prevention, risk assessment and risk management are critical to safer environments, it is important to have tools and studies to help achieve these goals.

## Data Availability

Publicly available datasets were analyzed in this study. This data can be found here: https://www.dropbox.com/s/25sr0bnya9vmwhq/CCVSFb.xls?dl=0.

## Ethics Statement

This study was carried out in accordance with the recommendations of the “UDLAP Research Committee” with written informed consent from all subjects. All subjects gave written informed consent in accordance with the Declaration of Helsinki. The protocol was approved by the “UDLAP Research Committee.”

## Author Contributions

All authors contributed, to varying degrees, in each of the following: conception of the study, methodology and formal analysis, writing of the manuscript, review of form and content, and approval of the final version of the manuscript. In addition, JP-C was in charge of the project administration and supervision.

## Conflict of Interest Statement

The authors declare that the research was conducted in the absence of any commercial or financial relationships that could be construed as a potential conflict of interest.
